# Evaluation of a Lipid Profile in Normoglycemic and Pre-diabetic Saudi Office Employees in Makka Region: A Case-Control Study

**DOI:** 10.7759/cureus.57608

**Published:** 2024-04-04

**Authors:** Mohamed M Nour Eldein, Abdullatif T Babakr

**Affiliations:** 1 Biochemistry, Umm Al-Qura University, Makka, SAU; 2 Medical Biochemistry, Umm Al-Qura University, Makka, SAU

**Keywords:** sedentary behavior, lipid profile, lifestyle, dyslipidemia, diabetes

## Abstract

Background: Diabetes mellitus (DM) poses a significant health challenge worldwide. The impact of a sedentary lifestyle in predicting and managing complications of diabetes represents an urgent need for health strategies. The objective of this study was to evaluate the lipid profile among normoglycemic and prediabetic Saudi office workers.

Methods: The research was a case-control study carried out in Makkah al-Mukarramah (Kingdom of Saudi Arabia, KSA). Seventy-five office worker volunteers between the ages of 19 and 45 years were recruited for the study. The participants were divided into two groups: a control group of non-diabetic normal subjects (NGT) and prediabetic subjects with impaired fasting plasma glucose and/or impaired glucose tolerance (IGT), based on the American Diabetes Association recommendations. Measurements of glucose, hemoglobin A1C (HbA1C), total cholesterol (TC), triglycerides (TG), high-density lipoprotein (HDL), and low-density lipoprotein (LDL) were performed using standard procedures and commercial kits. Statistical analysis was performed to compare the lipid profile in the two groups, and a P-value of <0.05 was considered statistically significant.

Results: A proportion (58.7%) of the office workers are prediabetics; prediabetic office workers had higher total cholesterol compared to the control group (p < 0.05). Triglyceride levels were higher in office workers with prediabetes compared to the normoglycemic group (p < 0.05). LDL levels were elevated in the prediabetic office workers compared to the control group (p < 0.05).

Conclusion: Office employees with prediabetes exhibit elevated levels of cholesterol, triglycerides, and LDL. The disturbance in lipid profile may be linked to impaired glucose tolerance in individuals with a sedentary lifestyle, such as office workers.

## Introduction

Diabetes mellitus (DM) is a global health problem; the number of people with diabetes is projected to reach 366 million in the year 2030 [1]. Most of the increase will occur in developing countries where it is expected to rise by 150%, and in these countries, individuals in the prime of their lives are specifically impacted by diabetes. In these nations, 25% of all diabetic adults are below the age of 44. Conversely, in developed countries, only 8% of adults with diabetes fall into this age bracket [2].

In 2003, the Expert Committee on the Diagnosis and Classification of Diabetes Mellitus recognized an intermediate group of subjects whose glucose levels, although not meeting the criteria to be diagnosed as diabetics, were too high to be considered normal [3]. These persons were defined as having impaired fasting glucose (IFG) (fasting plasma glucose (FPG) levels = 100 mg/dL to 125 mg/dL) or impaired glucose tolerance (IGT) (2-h values in the oral glucose tolerance tests (OGTT) of 140 mg/dL to 199 mg/dL). These individuals have been referred to as having prediabetes; the term indicates a relatively high risk for the future development of diabetes [4].

Long time spent in a sedentary lifestyle (sitting or lying with low energy expenditure) is associated with an increased risk for type 2 diabetes mellitus (T2DM) and cardiovascular diseases (CVDs) [5]. Recent studies showed that sedentary behaviors are linked to increased risk of mortality from CVDs, diabetes, metabolic syndrome, and impaired glucose and lipid metabolism [6]. Diabetes therapy is not only about lowering glucose but also about the overall reduction in the risk factors for diabetic complications, which includes the control of blood lipids. This requires lifelong care and management. There is an ongoing necessity to conduct studies that investigate the lipid profile in communities characterized by sedentary lifestyles.

Current guidelines recommend at least 30 minutes of moderate- to vigorous-intensity physical activity (MVPA) on most days to prevent T2DM and CVDs [7]. However, most office workers spend much of their day in environments that not only limit their physical activity but also require them to sit for prolonged periods.

Saudi Arabia has witnessed great lifestyle changes over the past few decades, and sedentary lifestyles are becoming prevalent among Saudi children and youth [8]. Data from previous studies indicate that 71% of young people in the Kingdom do not engage in physical activity of sufficient duration and frequency [9]. Being physically active is not only important in treating T2DM but also in decreasing the risk of developing the disease; it can be considered an important preventive measure [10].

It has been shown that higher levels of physical activity decrease the incidence of and mortality from CVDs. Exercise promotes a significant change in the cardiometabolic profile [11]. Lifestyle modifications by improving dietary habits, and enough duration and frequency of physical activity is the first-line approach to reducing overweight and related cardiovascular risks [12].

Office workers, in addition to physical inactivity, are subjected to occupational stress. Occupational stress is caused due to the imbalance between job demands and an individual’s ability, and it has been implicated as an etiology for CVDs; some cardiovascular risk factors were also more frequent in managers [13].

Dyslipidemia (increased levels of serum total cholesterol, triglycerides, and low-density lipoprotein (LDL) cholesterol and low levels of high-density lipoprotein (HDL) cholesterol) has been long recognized as a major biochemical event predisposing to atherogenicity; it is a major modifiable risk factor for CVDs [14].

Increases in the levels of serum total cholesterol and lipids accompanied by changes in lifestyle is an important health concern. The relationship between lifestyle factors such as dietary habits and physical activity on the one hand and between serum lipid and lipoprotein levels on the other hand is therefore of considerable interest. The lifestyle factors related to the development of dyslipidemia have to be identified.

Although effective pharmacological treatments for hyperlipidemia have been developed and used worldwide in congenital heart disease (CHD) management, long-term use of cholesterol-lowering drugs carries both costs and risks [15]. Target cholesterol levels as stated in various guidelines could be achieved by lifestyle changes, including weight reduction, diet, and increased physical activity; regular physical activity has been shown to reduce very-low-density lipoprotein (VLDL) levels, raise HDL cholesterol, and, to a lesser extent, lower the LDL levels and reduce insulin resistance [16].

The achievement of recommended levels of serum cholesterol, triglycerides, and lipoproteins is beneficial, especially in cases at risk of developing cardiovascular diseases such as prediabetics and individuals with low physical activity such as office workers. Studies that focus on dyslipidemia as a modifiable risk factor for the development of cardiovascular diseases continue to be crucial. The aim of the present study was the assessment of disturbance in the lipid profile in normoglycemic and prediabetic Saudi office workers.

## Materials and methods

The research was a case-control study carried out in Makkah al-Mukarramah (Kingdom of Saudi Arabia, KSA). The study protocol and procedures received approval from the Biomedical Ethics Committee (No. 41), Faculty of Medicine, Umm Al-Qura University, Makkah, KSA.

The study recruited volunteers from both normoglycemic and prediabetic office workers in the holy city of Makkah. Office workers are participants who spend usually eight hours or more sitting in their offices without considerable physical activity; those subjects were recruited from different governmental and private sectors in Makkah. We calculated the sample size using the following formula:

n1 = (σ1 + σ2 / K) (Z1-α/2 + Z 1-β)2 /Δ2 , and (K = n2/n1 = 1),

Where:

n1, n2 = sample size of groups, σ1, σ2 = variance of means, α = probability of type 1 error (0.05), β = probability of type II error (0.2), Z = critical Z value for a given α or β, Δ = absolute difference between two means, and K = ratio of sample size (1).

The minimum sample size according to this formula was found to be 40 participants. A total of 75 adult male volunteers between the ages of 19 and 45, who willingly agreed to participate, were selected for the investigation. These participants were provided with detailed information about the study's purpose, potential risks, and ethical considerations, and they signed a consent form. The population under study was then divided into two groups based on the guidelines set forth by the American Diabetes Association (ADA) for diagnosing diabetes and classifying glucose tolerance [3]: Group I is the control group of non-diabetic normal subjects (NGT) and Group II is the prediabetic subjects with impaired fasting plasma glucose and/or impaired glucose tolerance (IGT).

Participants who were excluded from the study were those with overt diabetes, heart diseases, and aged less than 18 or more than 45 years old.

Blood samples were collected in plain tubes for all participants using standard vein puncture procedures, left for 30 minutes, and then centrifuged for 15 minutes at 3000 rpm, and the serum samples were obtained. The tubes were properly labeled and sent directly to the biochemistry laboratory. Serum samples intended for long storage were kept at -80 °C up to the date of analysis.

Glucose, HbA1c, total cholesterol (TC), triglycerides (TG), HDL, and LDL levels were assessed by utilizing established protocols and commercially accessible kits within a completely automated system (COBAS Integra 400 Plus).

The reagents used for the measurements were CHOD-PAP for TC, GPO-PAP for TG, LDL-C plus second generation for LDL-cholesterol, and HDL-C plus third generation for HDL-cholesterol. All assays were done following the recommended procedures for instrument operation, calibration, quality control, and assay guidelines. The instrument was calibrated using a calibrator for automated systems (Roche Diagnostics) for glucose, TC, and TG and a calibrator for automated systems lipids (Roche Diagnostics) for LDL-cholesterol and HDL-cholesterol. Results were expressed for all parameters in mg/dl, except for HbA1c, where it was expressed as a percentage of glycosylated hemoglobin [17].

Statistical analysis

Descriptive statistics was used for the tabulation of the studied parameters in the two groups, and then the independent t-test was used to compare the levels of all metabolic parameters between the two groups to examine the effect of office working on the lipid profile. A P-value < 0.05 was considered statistically significant. All statistical methods were performed using IBM SPSS Statistics for Windows, version 27.0 (released 2020, IBM Corp., Armonk, NY).

## Results

**Figure 1:**
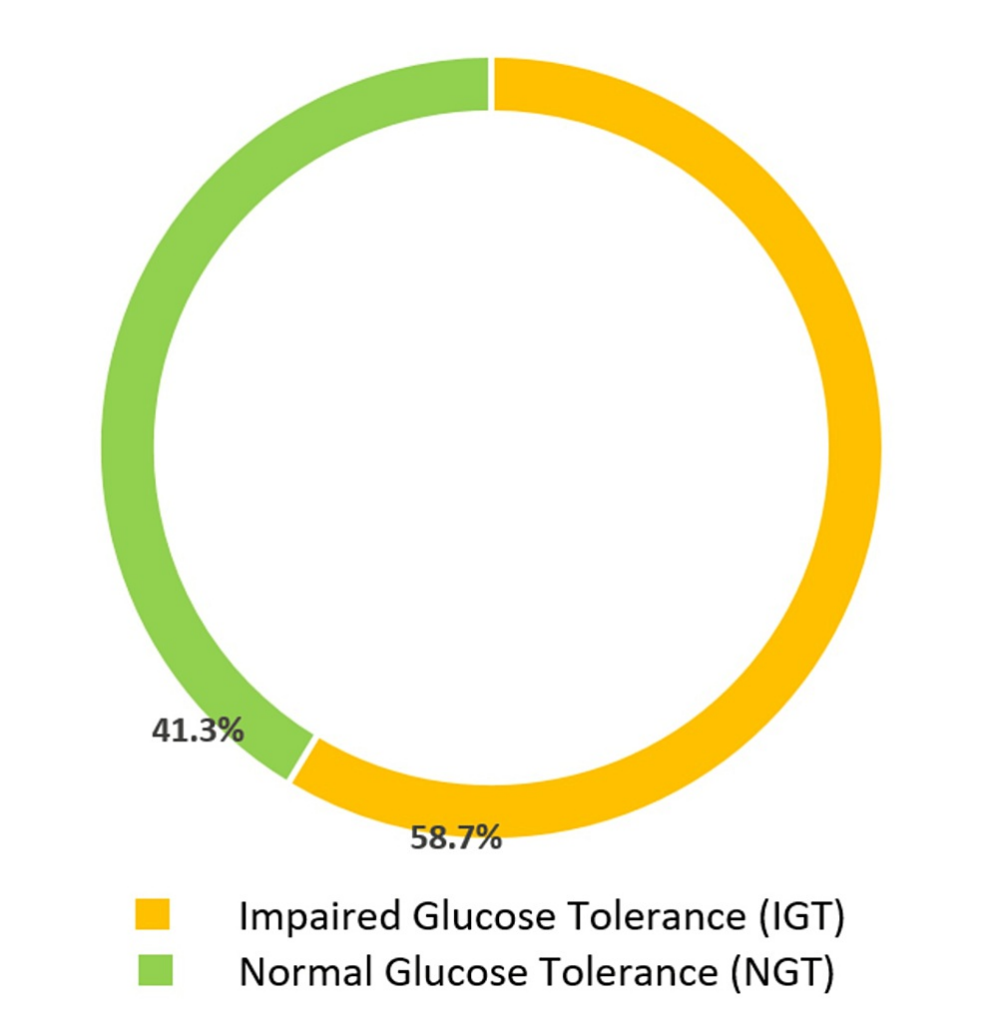
Percent of cases of normal glucose tolerance (NGT) and impaired glucose tolerance (IGT) among office workers

Seventy-five office worker participants were involved in the present study; 31 (41.3%) were included in the normal glucose tolerance group (NGT), and the second group of impaired glucose tolerance included 44 office workers (58.7%), as shown in Figure 1.

Our findings indicate that there is no statistically significant disparity in the body mass index (BMI) between office workers and the general population, as revealed by anthropometric measurements and shown in Table 1.

**Table 1:** Age, BMI, HbA1c, and lipid profile of the study groups. FBS: fasting blood sugar; 2hr pp: two-hour postprandial blood sugar, BMI: body mass index, HbA1c: hemoglobin A1C, HDL-C: high-density lipoprotein cholesterol, LDL-C: low-density lipoprotein cholesterol Results are expressed as mean ± standard deviation (SD).

	Normal glucose tolerance of office workers (N = 31)	Prediabetic office workers (N = 44)	P-value
Age (Yrs)	35 ± 9.1	40 ± 10.5	NS
BMI (Kg/m^2^)	27.9 ± 4.2	30.9 ± 4.4	<0.05
HbA1c (%)	5.0 ± 0.61	6.9 ± 1.3	<0.001
FBS (mg/dl)	90.1± 10.1	125.6 ± 12.2	<0.001
2hr pp (mg/dl)	106 ± 22.4	169.6 ± 11.6	<0.001
Cholesterol (mg/dl)	245 ± 56.4	270 ± 51.6	<0.05
Triglycerides (mg/dl)	189 ± 95.8	236 ± 118	<0.05
HDL-C (mg/dl)	55.0 ± 18.4	56.8 ± 17.4	NS
LDL-C (mg/dl)	144 ± 23.3	158 ± 32.7	<0.05

The means of HbA1c was (5 ± 0.61%) in the NGT group, while an increased level was observed in the IGT group office workers (6.9 ± 1.3%; p < 0.001), as shown in Table 1.

**Figure 2:**
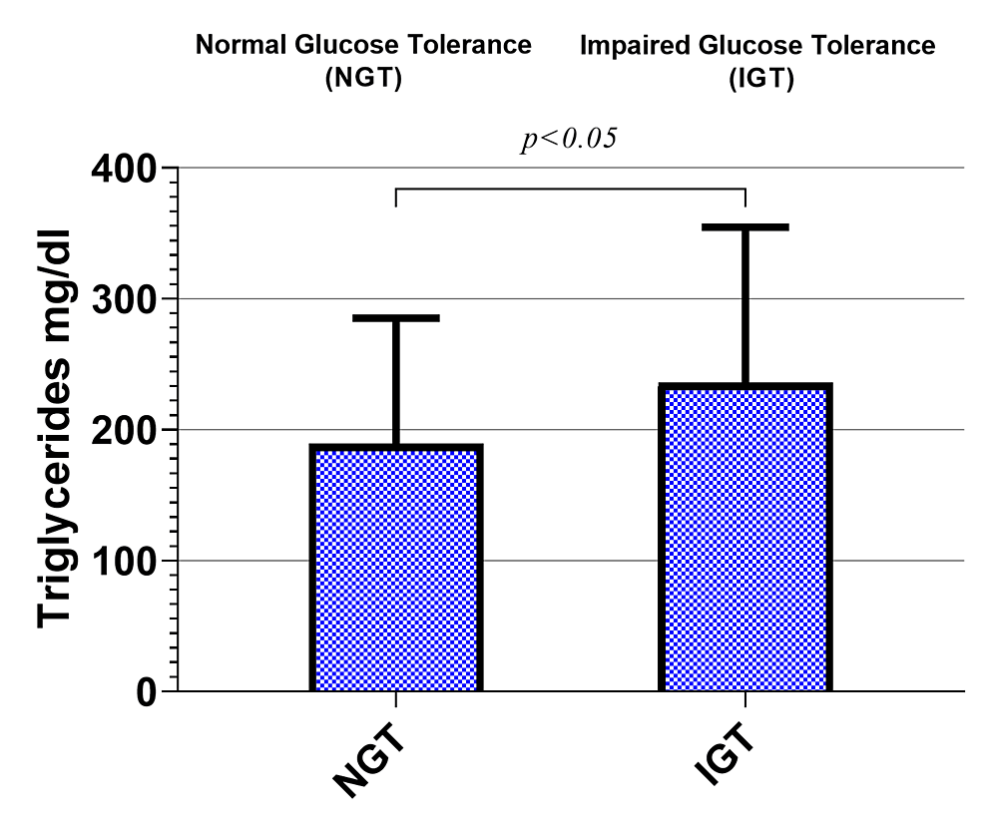
Triglyceride levels in the study groups.

A significant increase was reported in the triglycerides of office workers (236 ± 118 mg/dl) compared to the normoglycemic office workers (189 ± 95.8 mg/dl; p < 0.05), as shown in Figure 2.

**Figure 3:**
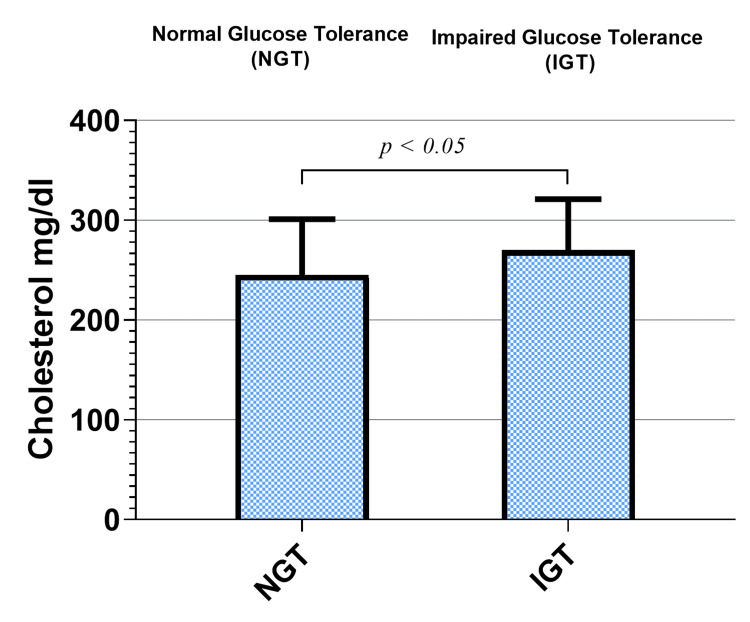
Cholesterol levels in the study groups.

Levels of TC increased in the prediabetic group compared to the normoglycemic one. TC increased significantly in the prediabetic office workers (270 ± 51.6 mg/dl) compared to the NGT group (245 ± 56.4 mg/dl; p < 0.05), as shown in Figure 3.

**Figure 4:**
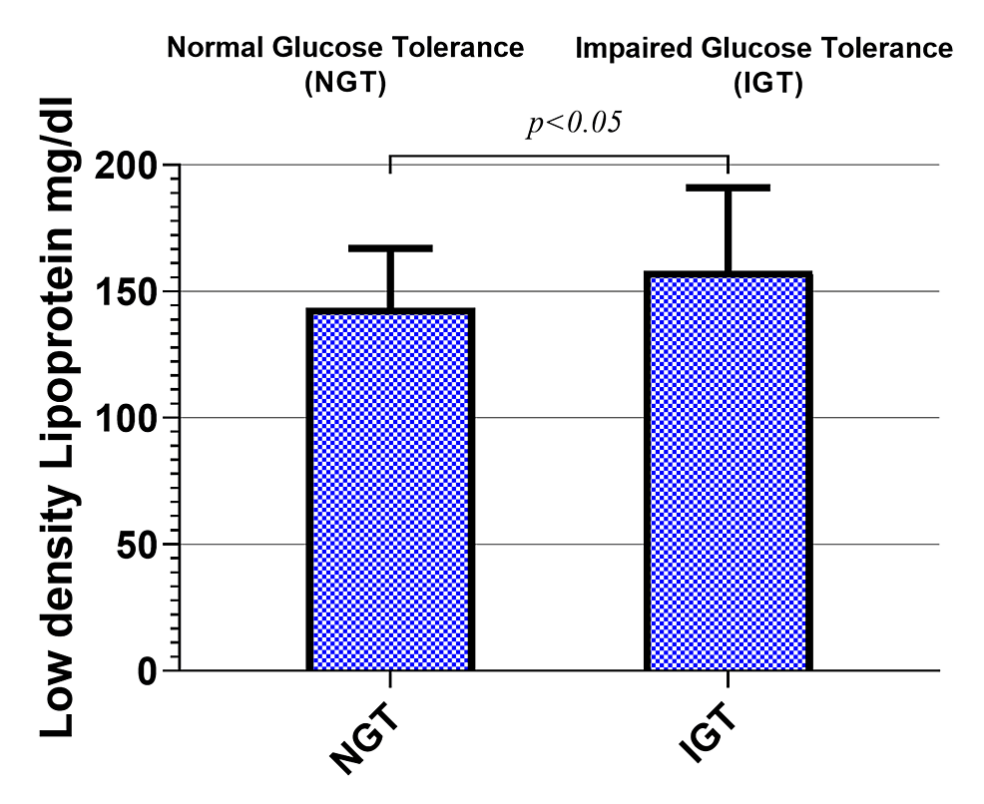
Low-density lipoprotein levels in the study groups

Similar to the levels of TC, the mean levels of LDL were elevated in prediabetic office workers compared to the NGT group, where it was 158 ± 32.7 and 144 ± 23.3 mg/dl in the two groups, respectively (p < 0.05), as shown in Figure 4.

## Discussion

According to the findings of this study, 58.7% of the office workers had impaired glucose tolerance (IGT) (Figure 1). This study offers insights into the relatively high frequency of prediabetes cases among Saudi people working in office environments; prediabetes was more commonly observed in older men and those with a higher BMI. Earlier research revealed that prediabetes increases the risk of CVDs [18]. In 2014, a concerning finding emerged from a study conducted on households in Saudi Arabia, revealing that approximately 50% of the adult population exhibited some form of impaired glucose metabolism. This study further disclosed that there was a notable prevalence of diagnosed diabetes, reaching a staggering 25.4%, while an additional 25.5% were found to have prediabetes, indicating a high risk of developing the disease in the future [19]. Studies on the epidemiology of diabetes in a different Saudi community have subsequently validated these numbers [20]. According to a recent investigation conducted on elite football players, it was found that the occurrence of prediabetes among this group was recorded at 7.9%. By contrast, when compared to a group of nonathlete individuals with similar characteristics, the prevalence of prediabetes was notably higher at 18.78%. [21]. The outcomes of this study contribute to the mounting evidence of the worrisome prevalence of glucose intolerance in the Saudi population.

In comparison to the prevalence data on a global scale, the current study reveals a notably high prevalence of prediabetes. The International Diabetes Federation (IDF) reports indicate that the Middle East and North Africa region is experiencing a rapid increase in the prevalence of prediabetes; 2019 saw the highest incidence of diabetes, with a prevalence rate of 12.2%. This worrisome trend is accompanied by considerable morbidity and fatality rates related to the condition [20]. The reason why we observed a high prevalence of prediabetes in our study is that we specifically identified prediabetes in office workers by measuring their HbA1c levels, as well as their fasting blood glucose (FBG) and two-hour post-prandial blood glucose (2hrPP). These measurements are considered to be more accurate indicators of prediabetes. Other studies that have used HbA1c criteria have also reported a higher prevalence of prediabetes. It is not surprising to see such a high prevalence of prediabetes in office workers, given the high rates of obesity and physical inactivity among this population [22,23].

The occurrence of undiagnosed diabetes was substantial and on the rise [24]. The concept of ensuring universal health coverage takes precedence, encompassing the integration of primary, secondary, and tertiary healthcare levels, along with the implementation of existing action plans that are crucial. Both age and obesity have been widely recognized as established risk factors for both DM and prediabetes. These two factors have been extensively studied and shown to significantly increase the likelihood of developing these conditions. Age, with its associated physiological changes and decreased metabolic efficiency, has been found to contribute to the onset of DM and prediabetes. Similarly, obesity, characterized by excessive accumulation of body fat, has been linked to insulin resistance and impaired glucose metabolism, making individuals more susceptible to both DM and prediabetes. The relationship between age, obesity, and the development of these conditions has been extensively documented in numerous scientific studies, reinforcing the importance of addressing these risk factors in preventive healthcare strategies. The carbohydrate challenge imposed against metabolic homeostasis in the older population causes average FBG levels to increase with age [25].

The results of this study showed that office workers who had prediabetes had significantly higher levels of various CVD risk factors, such as dyslipidemia, compared to their normoglycemic peers. These findings align with previous research that has consistently demonstrated the increased risk of CVD associated with DM even before clinical symptoms appear. It is well-established that CVD, stroke, and peripheral vascular disorders are all considered macrovascular diseases that are closely linked to T2DM. In addition, the traditional risk factors for CVD, including dyslipidemia, obesity, and hypertension, are particularly prevalent among office workers with prediabetes, as evidenced by numerous studies [26]. The pathophysiological changes observed in this scenario involve a decrease in the beta cells, dysfunction of the endothelial cells, increased stiffness of the arteries, heightened breakdown of lipids, and cytokine dysregulation. These alterations contribute to the development and progression of the condition [2,27].

The significance of dyslipidemia as a predictor for prediabetic office workers was the main focus of the current study, which aimed to assess the association between prediabetes and lipid parameters. In addition, the study aimed to analyze the strength of association of different parameters and account for the potential influence or confounding effect of other demographic and non-modifiable risk factors. These findings are presented in Table 1. Previous research has extensively explored the relationship between dyslipidemia and prediabetes, with evidence suggesting that the presence of dyslipidemia is associated with an increased risk of developing prediabetes and progressing to T2DM [28]. In addition to experiencing rapid growth and more severe complications related to diabetes, our study found a significant correlation between prediabetes in office workers and various lipid parameters, as outlined in Table 1. This aligns with previous findings in different populations, highlighting that any form of dyslipidemia, whether individually or in combination, particularly elevated levels of LDL-C and triglycerides, are linked to a higher likelihood of prediabetes in office workers.

In the present study, a significant increase was observed in the triglyceride levels of prediabetic office workers compared to the normoglycemic group. This finding suggests that a sedentary lifestyle may contribute to dyslipidemia in individuals with impaired glucose tolerance.

Serum lipid profile parameters are commonly assessed as a part of CVD prevention initiatives, following prevailing guidelines for diabetes and prediabetes [29]. However, a complete evaluation of dysglycemia is seldom sought and usually relies on the evaluation of fasting glucose alone, resulting in underdiagnosis and an increased risk of diabetes and CVDs. In the current study, dyslipidemia was found in instances with IGT in office workers. Moreover, greater levels of TC and LDL were found in office workers with prediabetes, as shown in Figure 3 and Figure 4.

Previous research findings suggest that a significant proportion of the youth population in the kingdom, approximately 71%, do not partake in physical activities that meet the recommended duration and frequency [9]. Engaging in regular physical activity is not only crucial for managing T2DM but also plays a vital role in reducing the likelihood of developing this condition. Therefore, it can be regarded as a significant preventive measure to combat the disease [10].

Our study has both strengths and limitations. The primary strength of the present study is that it may be considered the first in-depth investigation to compare the lipid profile characteristics of NGT and IGT groups among office workers. The fundamental drawback, which is shared by all such cross-sectional research, is that a relationship can only be proposed due to the small sample size; nevertheless, a cohort study is required to verify any suggested associations. As a result of our findings, we propose universal screening for glucose tolerance of dyslipidemic individuals, especially in groups of similar sedentary lifestyles.

Early identification of prediabetes among office workers will aid in the implementation of dietary and lifestyle measures, as well as medication where needed, to avoid or postpone the development of overt T2DM, and therefore the associated higher risk of CVDs. Due to the significant prevalence of T2DM in the community, it is crucial to develop and implement comprehensive screening and prevention initiatives for prediabetes among office workers. These programs should be carefully designed, considering the known predictors of prediabetes, and should also include screening for prediabetes in individuals with dyslipidemia compared to other populations at risk.

## Conclusions

The current study looked at the lipid profile parameters in normoglycemic and prediabetic office workers. Prediabetes was found to be associated with dyslipidemia. Moreover, prediabetic office workers exhibited higher levels of CVD risk variables, particularly higher levels of TC, TG, and LDL. The present study suggests that sedentary behavior may lead to dyslipidemia in people with poor glucose tolerance. The relevance of physical exercise in reducing impaired glucose tolerance that can lead to T2DM was emphasized.
